# Igh Locus Polymorphism May Dictate Topological Chromatin Conformation and V Gene Usage in the Ig Repertoire

**DOI:** 10.3389/fimmu.2021.682589

**Published:** 2021-05-18

**Authors:** Amy L. Kenter, Corey T. Watson, Jan-Hendrik Spille

**Affiliations:** ^1^ Department of Microbiology and Immunology, University of Illinois College of Medicine, Chicago, IL, United States; ^2^ Department of Biochemistry and Molecular Genetics, University of Louisville School of Medicine, Louisville, KY, United States; ^3^ Department of Physics, University of Illinois at Chicago, Chicago, IL, United States

**Keywords:** Chromatin, B cell, immunoglobulin, VDJ recombination, VDJ repertoire

## Abstract

Vast repertoires of unique antigen receptors are created in developing B and T lymphocytes. The antigen receptor loci contain many variable (V), diversity (D) and joining (J) gene segments that are arrayed across very large genomic expanses and are joined to form variable-region exons of expressed immunoglobulins and T cell receptors. This process creates the potential for an organism to respond to large numbers of different pathogens. Here, we consider the possibility that genetic polymorphisms with alterations in a vast array of regulatory elements in the immunoglobulin heavy chain (IgH) locus lead to changes in locus topology and impact immune-repertoire formation.

## Introduction

The adaptive immune response has evolved to recognize pathogens using antigen-specific receptors expressed on B and T lymphocytes. Two identical immunoglobulin (Ig) heavy chains (IgH) and two identical light chains (Igκ or Igλ) constitute the B-cell receptor (BCR). The two lineages of T cells are distinguished by the type of T-cell receptor (TCR) expressed. TCRαβ is encoded by the Tcra and Tcrb loci, whereas TCRγδ is encoded by the Tcrg and Tcrd loci. Developing B and T cells undergo an ordered set of DNA rearrangements termed V(D)J recombination, using RAG recombinase (RAG1/2) and thereby creating a diverse repertoire of antigen receptors (1). The assembly of antigen receptors involves the juxtaposition of variable (V), diversity (D) and joining (J) gene segments into a V gene exon that encodes the antigen binding domain of antigen receptors. However, there are several barriers which must be overcome to enable a suitably diverse Ig repertoire to emerge. Many of the concepts discussed here are applicable to TCR loci.

**Figure 1:**
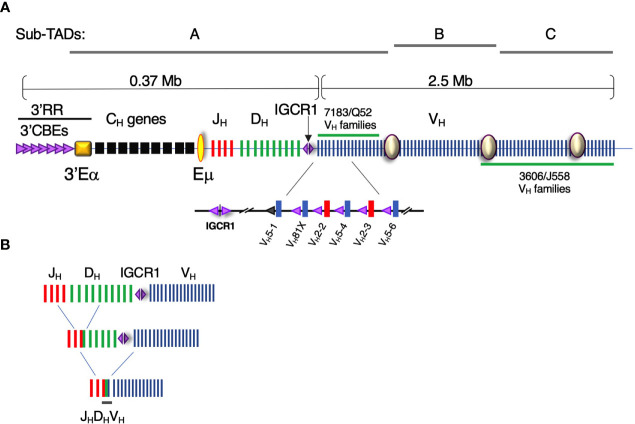
The Igh locus contains ~100 V_H_ gene segments over an almost 3Mb genomic interval. **(A)** (Upper panel) Diagram of the Igh locus indicating V_H_, D, J_H_, and C_H_ exons and regulatory elements (not to scale). The intronic Eμ and 3′Eα super-enhancers and intergenic control region 1 (IGCR1), composed of two divergent CBEs, are critical regulatory elements. CBE orientation is indicated by (purple) triangle direction. The 3′ regulatory region (3′RR) is a composite of nine CBEs located at the 3′ boundary of the Igh locus adjacent to 3′Eα super-enhancer. Sites I, II, and III (purple circles) anchor the sub-topologically associating domain (Sub-TADs) A, B, and C. The V_H_S107 family along with nine smaller V_H_ families comprise the intermediate V_H_ segments. The interspersed distal V_H_ gene segments are composed of the V_H_J558 and V_H_3609 families and are located at the 5’ end of the locus. (Lower panel) The V_H_7183 (blue bars) and V_H_Q52 families (red bars) are located at the D_H_J_H_-proximal end of the locus. Each D_H_J_H_ -proximal V_H_ exon is paired with a recombination signal sequence (not shown) and a CBE (purple triangle). The CBE associated with VH5-1 exon is non-functional (gray triangle). VH81X (V_H_5-2) is the second V_H_ exon gene relative to IGCR1. **(B)** Schematic of the stepwise process of V(D)J recombination. D_H_-J_H_ rearrangement precedes V_H_-D_H_J_H_ recombination.

Formation of a diverse Ig repertoire is critically dependent on proficient pro-B and pre-B cell function since it is in these cells that IgH and IgL chain genes are assembled through V(D)J recombination, respectively. V(D)J recombination requires that antigen receptor genes undergo ordered rearrangement with D_H_ to J_H_ joining preceding V_H_ to D_H_J_H_ recombination (**Figure 1A**). There are ~100 functional Igh locus V_H_ gene exons that must recombine with one rearranged DJ_H_ element, that is assembled from one of 8-12 D_H_ and one of 4 J_H_ gene segments in C57BL/6 mice (**Figure 1**) (1). The introduction of RAG dependent DNA breaks at recombination signal sequences (RSSs) adjacent to each rearranging gene segment initiates Igh gene assembly (1). RAG1/2 loads at the recombination center (RC) situated in the region spanning Eµ and the most 3’ D_H_ segment, DQ52 (2). RAG1/2 has been proposed to track from the RC to locate a suitable RSS for synapsis and DNA cleavage (3).

## The V_H_ Gene Usage Conundrum

The number of V, D, and J gene segments and the availability of those segments for rearrangement determines the composition and complexity of antigen receptor repertoires. The Igh locus is quite large in linear genomic distance, extending 2.9 Mb and containing ~100 functional V_H_ gene segments. V gene usage is only quasi-random in the pre-selected Igh repertoire as V genes rearrange at very different intrinsic frequencies (4–11). In studies of V germline transcript levels, transcription factor (TF) binding, RSS quality, and the distribution of a variety of epigenetic marks each make a contribution, but no one variable, or combination of variables, fully accounts for unequal V gene usage (4–6, 8, 12). Although the V gene accessibility hypothesis (13–15) offered an attractive model to explain V gene usage, recent studies have made plain that V gene accessibility is a necessary but insufficient condition for participation in V->DJ rearrangement (16, 17). Therefore, the factors underpinning unequal V gene rearrangement frequencies remain to be determined.

It is important to note that the potential contribution of Ig haplotype diversity in these processes has been underappreciated (18). Despite the fact that the Ig loci of natural outbred organisms are known to be extremely diverse, much of our understanding of the mechanisms dictating V(D)J recombination have come from studies of inbred models. However, even in inbred models, it has been demonstrated that Ig genetic diversity is more extensive than initially appreciated, in many cases mirroring (or even exceeding) what has been observed in human populations and other more outbred organisms (19–28). In mouse, for example, a comparison of germline V_H_ sequences between C57BL/6 and BALB/c revealed surprisingly little overlap in the germline repertoires of these two strains. Of the 99 C57BL/6 and 164 BALB/c V_H_ alleles compared, only 5 were found to be identical (23), likely the result of both allelic sequence divergence and structural variation associated with differences in V gene content between the two strains. Extended comparisons of V_H_ germline alleles across additional inbred wild-derived strains, thought to represent diverse mouse sub-species origins, revealed even greater diversity, suggesting that Ig germline variation across commonly used mouse inbred strains is likely to be vast (24). Similar inter-strain diversity has been observed within the mouse D_H_ gene loci as well (19, 20). These and other data clearly demonstrate the presence of extensive polymorphism within the Igh locus, and highlight the potential influence of both sequence diversity and Ig gene segment number as significant contributors to Ig repertoire diversity.

There is a growing body of evidence supporting the potential impact of genetic polymorphism on V(D)J recombination. First, multiple studies of the naïve repertoire in human monozygotic twins have demonstrated that V_H_, D_H_, and J_H_ usage is highly heritable (29, 30). Second, genetic variants within the Ig loci associate with variation in gene usage observed between individuals, including examples of Ig gene coding and non-coding single nucleotide polymorphisms (SNPs) (31, 32), as well as large structural variants (32–34). Here we examine the confluence of V_H_ gene identity with locus polymorphism and locus architecture as determinants for V_H_ gene usage in V(D)J recombination and ultimately the diversity of the preselected Ig repertoire.

## Igh Locus Architecture and Contraction Are Implicated in Repertoire Diversity

It is essential that all V_H_ genes achieve spatial proximity with the RC located at the Eµ-D_H_J_H_ domain to produce a fully representative Ig repertoire (**Figure 1B**). In C57BL/6, V_H_ exons are segregated into three clusters, the proximal, intermediate and distal V_H_ regions, that collectively span ~2.4 Mb. Recent studies have established that chromosomes are folded into hierarchical domains of various length scales. Chromosomes are nonrandomly located in nuclei within chromosomal territories (35, 36) which are subdivided into chromosomal compartments (36) that are further partitioned into topologically associating domains (TADs) (36, 37). TADs are zones in which intraregional interactions are more frequent than those traversing the boundaries between TADs (37–39). TAD organization reflects the functional partition of chromatin regions by transcriptional activity (37, 39), histone modifications (37–40), and replication timing (41) implying a link between function and genome structure.

The Igh locus is contained within a 2.9-Mb TAD in pro-B cells (42). 5C studies demonstrate that the murine Igh TAD is subdivided into two highly structured sub-TADs A and C, corresponding to the D_H_-proximal and D_H_-distal V_H_ gene families, respectively, while the less structured sub-TAD B includes the intermediate V_H_ gene segments (42). Correspondingly, live pro-B cell imaging indicates that Igh locus topology is organized as a series of three large, intermingled chromatin loops anchored close to the DJ_H_ RC, that provide comparable access between distal V_H_ gene segments with rearranged 3’ D_H_J_H_ (43). Hence, Igh locus topology is best described as a series of three large chromatin loops that are anchored at sub-TAD boundaries.

## The Building Blocks of TAD Architecture: Loop Extrusion, CTCF and Enhancer-Promoter Contacts

TAD boundaries are frequently marked by CBEs in a convergent orientation (40, 44) which participate in loop extrusion (45, 46). The loop extrusion model posits that chromatin loops are formed when cohesin is loaded onto and reels in DNA in an ATP-dependent process (45–48). Architectural “stripes”, visualized within Hi-C maps (49) may form when one subunit cohesin stalls near a strong CTCF loop anchor while the second one slides along the chromatin to form multiple interactions. The extrusion model explains how enhancers can processively track along arrays of promoters separated by long genomic intervals (45, 46, 50) and has been proposed as the mechanism that enforces deletional CSR (51, 52) and creates Igh locus contraction during V(D)J recombination (52). However, while sharp TAD boundaries are lost upon CTCF inactivation, compartment organization as well as TAD-like globular chromatin domain structures are preserved in single cell experiments (53) and the impact of CTCF inactivation on the transcriptome is small (54). Cohesin has been shown to promote clustering of enhancer elements in 3D spatial hubs (55). Intra-TAD contacts between regulatory elements facilitated by cohesin loop extrusion can be stabilized by other mechanisms such as homo-dimerization of the structural regulator YY1 (56). The inter-relationship of loop extrusion and a putative promoter-enhancer interactome in the Igh locus remains largely undefined.

## Is Igh Locus Topology Configured by a Promoter-Enhancer Interactome?

New unpublished work from the Kenter group has identified highly transcribed V_H_ gene promoters and a series of novel enhancers (NEs) that are pro-B cell specific, are involved in anchoring Igh subTAD loops and influence Igh repertoire formation in pro-B cells through formation of a promoter-promoter-enhancer hub. This is an interesting proposition as hundreds of V_H_ exon promoters and newly recognized enhancers could participate in an intricate contact interactome that spatially organizes V_H_ segments within the previously defined large chromatin loops and defines access probability to the RC and DJ segments. The presence of intra-TAD promoter-promoter-enhancer interactomes has been documented in several genetic loci and in different developmental and differentiation systems. Here we consider evidence that enhancers and promoters initiate specific interactions in nuclear space and propose that this interactome influences repertoire diversity.

Multiple lines of evidence support the existence of enhancer interactomes in different genomic contexts (57). While some studies link chromatin contacts between regulatory elements to transcriptional activity (58), in other examples these contacts precede gene activation (59). Most prominently, enhancers in olfactory sensory neurons form a large inter-chromosomal hub (60). In other systems, super-enhancers, clustered arrays of enhancer elements in close spatial proximity that can span several kilobases and are linked to the regulation of cell-identity genes with high transcriptional activity (61, 62), are highly involved in the formation of specific chromatin contacts. Genome architecture mapping identified abundant three-way contacts between super-enhancers and highly transcribed chromatin regions beyond the pairwise interactions detectable by 3C techniques (58). Similarly, the enhancer elements within a super-enhancer and target promoters can cluster spatially to form a hub structure with simultaneous multi-way interactions as demonstrated by multi-contact 4C for the locus control region of the beta-globin locus (63).

## Intergenic Igh Polymorphism May Alter Enhancer and Promoter Function

**Figure 2:**
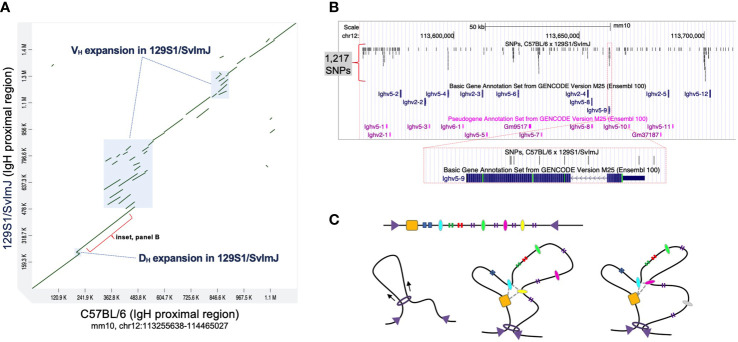
Comparison of the C57BL/6 and 129S1/SvlmJ Igh loci reveals significant polymorphisms. **(A)** A dot plot representing a sequence comparison of the proximal region of IgH (mm10, chr12:113255638-114465027) between the mouse strains 129S1/SvlmJ and C57BL/6; sequences from each strain were compared using MashMap (64, 65). Annotated boxes represent regions of complex structural variants, in which Ig gene segments vary in copy number between the two strains. Sequence identities within homologous regions (green diagonal lines) range between 84% and 98%. **(B)** A map of a 150 Kb region within the Igh proximal region (mm10, chr12:113562489-113712488). Shown are the positions of functional Ighv genes and pseudogenes, and single nucleotide polymorphisms (SNPs; indels not included) that differentiate the 129S1/SvlmJ and C57BL/6 haplotypes. In total, 1,217 SNPs are present, including variants in both coding and non-coding sequences, as illustrated in the inset panel centered on the functional gene Ighv5-9. SNPs in this region were identified by mapping the corresponding Igh sequence from 129S1/SvlmJ to mm10 using BLASR. **(C)** Top: Linear sequence of a chromatin neighborhood flanked by converging CTCF sites (purple triangles). Regulatory elements (ovals) and gene elements (vertical bars) are dispersed throughout the neighborhood. Left: Cohesin (ring) reels the chromatin fiber in to extrude a loop, facilitating interactions between regulatory elements. Middle: A promoter-enhancer interactome (gray dashed line) can confer locus structure with a distinct set of interactions. Right: Loss of association with the interactome results in topological changes and affects access to genetic elements.

When considering the Igh locus it is important to note that genetic differences between inbred strains extend beyond coding variation into intergenic regions. To date, the mouse IgH locus has only been fully characterized in C57BL/6, which, as we noted above, has served as the primary model for characterizing the functional regions and mechanisms that dictate V(D)J recombination. However, a partial assembly, including the proximal region of Igh in the 129S1/SvImJ mouse strain was published in 2007 (20). A comparison of these haplotypes revealed evidence of both local sequence conservation as well as divergence, including examples of structural variation and single nucleotide differences (20) (**Figure 2**). For example, several complex regions (**Figure 2A**) represent insertions of Ig genes in the 129S1/SvImJ strain that are absent in C57BL/6. In addition, the degree of sequence identity between these two strains varies considerably across the locus, with sequence identities ranging between 88% and 98%. Even in regions characterized by high degrees of homology between 129S1/SvImJ and C57BL/6, SNPs occur at relatively high densities in both coding and intergenic regions (**Figure 2B**). The impact of such inter-strain haplotype diversity on V(D)J recombination has not been investigated.

Elsewhere in the genome, deletions or mutations in enhancer sequences can lead to aberrant gene expression and disease phenotypes (66). Genome-wide association studies show that the vast majority of sequence variants associated with common diseases and traits are located in such non-coding parts of the genome (67). In line with long-range enhancer interactions discussed above, misregulation of target gene expression due to variation in enhancers can occur tens of kilobases away in linear sequence space (68).

Promoters and enhancers are characterized by a high density of sometimes overlapping TF binding sites. Active enhancers are established through the recruitment of TFs to those binding sites which opens chromatin (69). Mechanistically, TFs can mediate promoter–enhancer contacts in a variety of ways directly or indirectly through binding of additional factors and structural proteins (reviewed in (57)). For example, in mouse embryonic stem cells, deletion of KLF4 binding sites or KLF4 ablation results in reduced contact frequency in enhancer hubs and diminished expression of multiple target genes (70).

Single nucleotide changes in regulatory sequences can impact the affinity for TF binding (71). It can tip the balance in sites where different factors compete for the same space (72, 73) or regulatory regions have multiple functions (74). Since TF binding is essential for establishing an active enhancer, SNPs detected in Igh intergenic regulatory regions could potentially ensue in a cascade of downstream effects. They can alter TF binding in enhancers and promoters, impact long-range enhancer-promoter interactions, and thereby change the composition of promoter-promoter-enhancer interactomes (**Figure 2C**).

In this context it is significant that variation of intergenic CBE sequence can have a profound effect on V_H_ gene usage. V_H_ gene access to the RC was recently shown to be dependent on the quality of the flanking CTCF binding element (CBE) and related ability of the gene to loop with IGCR1 (16, 17). V_H_81X is the second gene in the locus and is most prominently used. V_H_5-1 is the most D_H_ proximal V_H_ gene in the locus and is very rarely used even though its promoter and recombination signal sequence are intact and similar to that found for V_H_81X. However, the quality of the flanking CBE for V_H_5-1 is poor and when replaced with a functional motif directs looping with IGCR1 and high frequency recombination. Thus, the quality of CBEs within the Igh locus highlights the importance of the integrity of similar regulatory sequences which can be altered by Igh polymorphisms.

## Conclusions

Genetic differences, such as those observed between 129S1/SvImJ and C57BL/6 (**Figures 2A, B**), when considered alongside observations that have been made for regulatory elements elsewhere in the genome, raise important questions about the potential for Ig genetic diversity to impact V(D)J recombination. First, there are numerous examples for which germline V_H_ variants have been shown to contribute to antigen specificity (75–79) and associate with disease and clinical phenotypes in the context of infection, inflammation, and vaccination (31, 32, 80–83). Second, both large structural variants and single nucleotide polymorphisms could modify key regulatory elements, such as CTCF sites and in promoters and enhancers, either through the disruption of these elements (e.g. sequence deletions or loss-of-functions SNPs) or the creation of novel elements (e.g. through sequence duplication or gain-of-function SNPs). In addition, structural variants could also be expected to change the spatial organization of interacting regulatory elements by increasing or decreasing the genomic distance between particular elements, or by changing their orientation. These modifications would in turn be expected to impact promoter/enhancer interactomes, lead to changes in the epigenetic landscape, and influence the overall locus architecture and TAD structure, and ultimately affect the selection of particular V_H_, D_H_, and J_H_ segments into the repertoire. We expect the discovery of such examples to continue as the inclusion of genetic variation in the study of repertoire diversity and dynamics becomes more commonplace.

## Data Availability Statement

Publicly available datasets were analyzed in this study. Sequence data and related gene annotations for the Igh locus in C57BL/6 were extracted from the mm10 genome reference assembly, available at https://genome.ucsc.edu. The sequence of the proximal Igh region of 129S1/SvlmJ was previously published by Retter et al. (20) available on GenBank under the accession number AJ851868.3.

## Author Contributions

Conceptualization, and Writing: AK, CW, and J-HS. Funding Acquisition, AK. All authors contributed to the article and approved the submitted version.

## Funding

This work was supported by grants to AK from the NIH (RO1AI121286, R21AI151892).

## Conflict of Interest

The authors declare that the research was conducted in the absence of any commercial or financial relationships that could be construed as a potential conflict of interest.
